# Mice expressing HLA-DQ6α8β transgenes develop polychondritis spontaneously

**DOI:** 10.1186/ar2023

**Published:** 2006-07-27

**Authors:** Jennifer L Lamoureux, Jane Hoyt Buckner, Chella S David, David S Bradley

**Affiliations:** 1 Department of Microbiology and Immunology, University of North Dakota School of Medicine and Health Sciences, Grand Forks, North Dakota, USA; 2 Benaroya Research Institute, Virginia Mason Medical Center, Seattle, Washington, USA; 3 Department of Immunology, Mayo Clinic College of Medicine, Rochester, Minnesota, USA

## Abstract

Relapsing polychondritis (RP) is a human autoimmune disease of unknown etiology in which cartilaginous sites are destroyed by cyclic inflammatory episodes beginning, most commonly, during the fourth or fifth decade of life. We have previously described collagen-induced polychondritis that closely mirrors RP occurring in young (6–8 weeks old) HLA-DQ6αβ8αβ transgenic Aβ0 mice, following immunization with heterologous type II collagen (CII).

We present evidence here that transgenic strains expressing the DQ6α8β transgene develop spontaneous polychondritis (SP) at the mouse equivalent of human middle age (4.5–6 months and 40–50 years old, respectively) and display polyarthritis, auricular chondritis and nasal chondritis – three of the most common sites affected in RP. Auricular chondritis in SP, like RP but unlike CII-induced polychondritis, exhibited a relapsing/remitting phenotype, requiring several inflammatory cycles before the cartilage is destroyed. Elevated serum levels of total IgG corresponded with the onset of disease in SP, as in RP and CII-induced polychondritis. No CII-specific immune response was detected in SP, however – more closely mirroring RP, in which as few as 30% of RP patients have been reported to have CII-specific IgG. CII-induced polychondritis displays a strong CII-specific immune response. SP also demonstrated a strong female preponderance, as some workers have reported in RP but has not observed in CII-induced polychondritis. These characteristics of SP allow for the examination of the immunopathogenesis of polychondritis in the absence of an overwhelming CII-specific immune response and the strong adjuvant-induced immunostimulatory influence in CII-induced polychondritis.

This spontaneous model of polychondritis provides a new and unique tool to investigate both the initiatory events as well as the immunopathogenic mechanisms occurring at cartilaginous sites during the cyclic inflammatory assaults of polychondritis.

## Introduction

Relapsing polychondritis (RP) is a human autoimmune disease of unknown etiology. RP is relatively rare, affecting 3.5–4/1,000,000 people, with a slight female preponderance [1]. Typical onset occurs during mid-life years with a median age of diagnosis at 46.6 years, although cases have been reported in newborns and in individuals over the age of 80 [2]. The most prominent manifestations of RP are auricular chondritis, polyarthritis and saddle nose deformation, with auricular chondritis being the most common presenting symptom [1,2]. Any other cartilaginous tissue may also be affected in RP, including the eyes, the trachea and the respiratory tract [2,3]. Other tissues can also be affected since some RP patients have demonstrated renal involvement and skin lesions [2,4]. Autoantibodies specific for type II, type IX and type XI collagen and for matrilin-1 [5-8] have all been detected in sera from RP patients, supporting RP as an autoimmune disease.

We have previously described a murine model of induced polychondritis that mimics many characteristics of RP [9,10]. Young mice (6–8 weeks of age) expressing the HLA-DQ6α8β molecule, either the DQ6α8β transgenic (tg) or DQ6αβ8αβ tg strains, develop polychondritis following immunization with an emulsion of type II collagen (CII) and Complete Fruend's Adjuvant. Induced polychondritis is characterized by a single acute inflammatory event, by auricular chondritis or by polyarthritis, followed by the destruction of the cartilage in both the peripheral joints and ears, and by pannus formation. Neither of the parental strains, DQ6αβ tg mice or DQ8αβ tg mice, develop polychondritis, although DQ8αβ tg mice do develop collagen-induced arthritis [9,10]. Hansson and colleagues [11] have also recently described a model of polychondritis, induced in DBA mice and in Lew congenic rats by immunization with bovine matrilin-1 in Complete Fruend's Adjuvant. In that model, there is inflammation of the trachea and respiratory tract, followed by their destruction; however, the ears, joints and other cartilaginous sites are not affected.

We present evidence that when mice expressing the DQ6α8β transgene and lacking both endogenous H2-A and H-2E expression, either DQ6αβ8αβ tg mice or DQ6α8β tg mice, reach middle age (between 4 and 6 months of age), they develop spontaneous polychondritis (SP), with inflammatory events occurring at the ears, the peripheral joints and the nose. Auricular chondritis in SP displayed a relapsing/remitting phenotype, more closely mirroring human RP than induced polychondritis. This model occurs in the absence of overt stimulation by an exogenous antigen such as CII, or adjuvant, or by artificially induced trauma to any of the sites of inflammation, and provides an excellent model that mirrors the human disease more closely than any previously described model of RP.

## Materials and methods

### Mice

The generation of mice expressing both HLA-DQ6αβ (DQA1*0103/DQB*0601) and HLA-DQ8αβ (DQA*0301/DQB*0302) transgenes in the absence of endogenous MHC class II expression (Aβ0) was previously described [9], and the mice were initially provided by Dr Chella David (Mayo Clinic, Rochester, MN, USA). Briefly, the transgenes were introduced into the H2^f ^background crossed with Aβ0 mice, resulting in tg mice expressing the DQ transgene and lacking both H2-A and H2-E expression. All breeding occurred in laminar flow isolation hoods to maintain a relatively pathogen-free environment. Retired breeders and mice aged greater than 6 months were moved to clean conventional rooms in the Center for Biomedical Research. Mice were given rodent chow and water *ad libitum*. All studies were approved by the University of North Dakota Institutional Animal Care and Usage Committee. Mice were bled retro-orbitally; the blood was spun for 10 minutes at 12,000 × *g *and the serum was decanted for analysis of IgG specificity.

### Total IgG levels

Total serum IgG levels were determined by ELISA as previously described [12]. Briefly, serum from experimental mice was diluted fourfold, 1/1,000–1/64,0000, in coating buffer (1 M Na_2_CO_3 _+ 1 M NaHCO_3 _+ 1 M NaN_3_), and 200 μl was added to Nunc Maxisorb microtiter plates in duplicate and incubated at room temperature for 2 hours. Plates were washed three times with PBS/Tween as in CII antibody, and then 100 μl/well goat anti-mouse immunoglobulin (Sigma-Aldrich, St Louis, MO, USA) was added and plates were incubated for 2 hours at 37°C. Plates were washed three times with PBS/Tween, and then 200 μl/well pNpp substrate (Sigma-Aldrich) was added and incubated for 1 hour at 37°C to visualize the reaction. The colorimetric change of each well was determined at an optical density of 405 nm on a Thermomax Microplate Reader (Molecular Devices, Sunnyvale, CA, USA), and the data were analyzed using the SOFTmax analytical software program (Molecular Devices) and Microsoft Excel.

### Type II collagen-specific antibody levels

One hundred microliters of collagen solution (Arthrogen-CIA^® ^mouse type II collagen, 5 mg/ml) diluted in 1 × collagen dilution buffer as per the manufacturer's instructions (Chondrex, Redmond, WA, USA) was added to Nunc Maxisorb microtiter plates (NNI, Rochester, NY, USA) and incubated overnight at 4°C. Plates were washed three times with PBS/0.5% Tween 20, were blocked by adding 1% BSA in PBS (blocking buffer) for 2 hours at room temperature and were then washed again three times with PBS/Tween. Experimental serum was diluted fourfold, 1/1,000–1/64,000, in blocking buffer/Tween, and then 200 μl was added to the plates in duplicate.

Titration of a high-titer CII-specific IgG-positive control and a CII-negative serum control were run with each plate. Sera were incubated for 2 hours at 37°C and were washed three times with PBS/Tween. CII-specific mouse IgG was detected with peroxidase-conjugated goat anti-mouse IgG (Sigma-Aldrich) for 1 hour at 37°C. The plates were again washed three times with PBS/Tween and the reaction visualized with pNpp substrate (Sigma-Aldrich). The colorimetric change of each well was determined at an optical density of 405 nm on a Thermomax Microplate reader, and the data were analyzed using the SOFTmax analytical software program (Molecular Devices) and Microsoft Excel.

### Detection of matrilin-1 antibodies

The presence of matrilin-1-specific IgG was determined in sera from representative SP mice, in sera from young and middle-aged naïve DQ6αβ8αβ tg mice and in sera from CII-immunized young DQ6αβ8αβ tg mice by western blot as previously described [8]. Briefly, matrilin proteins were extracted from bovine epiphyseal cartilages, were resolved by SDS-PAGE and were transblotted to polyvinylidene difluoride membrane (BioRad, Richmond, CA, USA) for western blot analysis. The western blot analysis was performed with mouse serum diluted 1:100 and probed with an alkaline phosphatase-conjugated goat anti-mouse IgG Fc (Jackson ImmunoResearch, Avondale, PA, USA). The blots were developed using 5-Bromo-4-chloro-3-indolyl phosphate/nitroblue tetrazolium.

### Histological analysis

Outer ears displaying clinical chondritis or outer ears from undiseased normal controls were surgically removed from mice anesthetized with Isofluorane (Phoenix Pharmaceutical Inc., St Joseph, MO, USA) at various time points in the course of disease. Ears were embedded in paraffin, were sectioned, were mounted onto slides and were stained with hematoxylin and eosin. Front and hind limbs were removed and dissected from euthanized mice at the conclusion of the experiment. Joints were decalcified and embedded in paraffin. Sections were cut, mounted and stained with hematoxylin and eosin. Sections of both ears and joints were also stained with Safranin O or Toludine Blue to determine the presence of mast cells. All sections were assessed in a blinded fashion by Dr Maryann Sens (Department of Pathology, UNDSMHS, Grand Forks, ND, USA) for histopathological changes and for cellular infiltration.

### Statistical analysis

Statistical analysis was carried out using the Mann–Whitney rank sum test for nonparametric tests. *P *< 0.05 was considered statistically significant unless otherwise noted.

## Results

### Spontaneous onset of polychondritis

**Figure 1:**
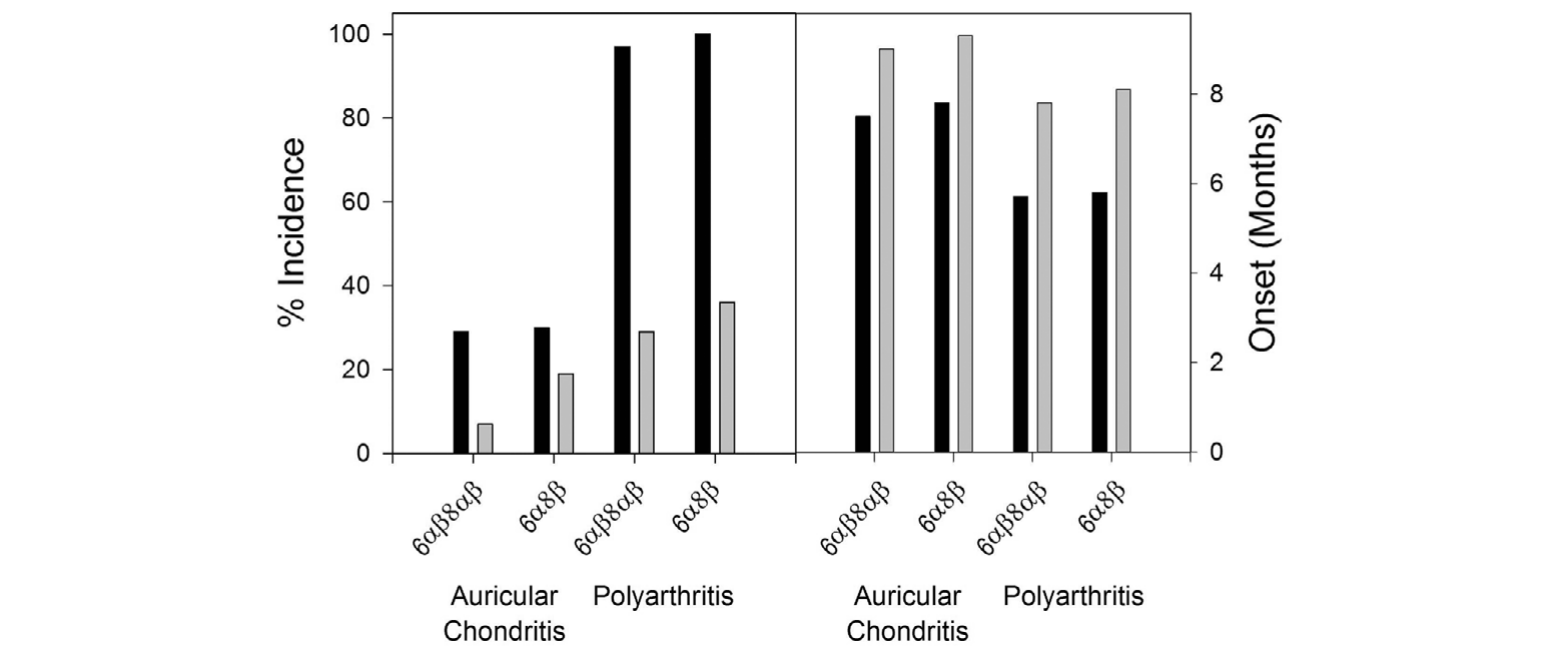
Incidence and onset of spontaneous polychondritis in female versus male DQ6α8β transgenic expressing mice. The onset of auricular chondritis and polyarthritis was determined by visual inspection and was confirmed by histological analyses as described in Table 1. Females (black shading) were more highly susceptible to both auricular chondritis and polyarthritis, and displayed clinical disease earlier than males (grey shading).

The incidence of SP in both DQ6αβ8αβ tg and DQ6α8β tg strains of mice is summarized in Table 1. DQ6αβ8αβ tg mice developed spontaneous polyarthritis at an incidence of about 80%, with 31% developing auricular chondritis. DQ6α8β tg mice developed spontaneous polyarthritis at an incidence of 76%, with 32% developing auricular chondritis (Figure 1). There was a strong female predisposition to developing SP in both DQ6α8β tg expressing strains: 3.8:1 and 4.4:1 female:male ratios in the DQ6αβ8αβ tg and DQ6α8β tg strains, respectively. Neither of the parental strains, DQ6αβ tg mice or DQ8αβ tg mice, developed SP. As we have reported elsewhere [13], however, DQ8αβ tg mice developed spontaneous polyarthritis at an incidence of 80% with, a few mice (<1%) displaying inflammatory events at other sites.

**Figure 2:**
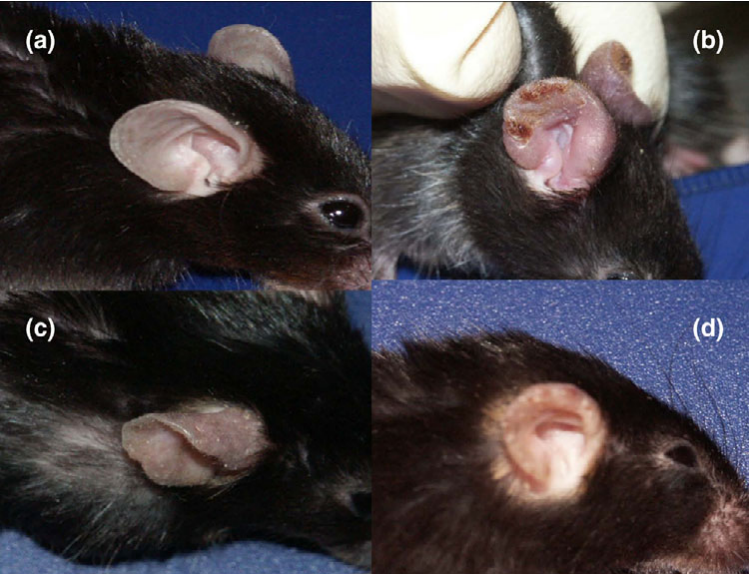
Representative photographs of relapsing/remitting auricular chondritis in DQ6α8β transgenic expressing mice. Ears were photographed at each of the clinical stages of auricular chondritis occurring in spontaneous polychondritis: **(a) **a normal, nonchondritic ear; **(b) **an acute chondritic ear in an inflamed cycle; **(c) **an acute chondritic ear in an noninflamed cycle; and **(d**) a chronically chondritic ear in which the cartilage has been destroyed.

The onset of spontaneous polyarthritis was observed from 4 to 11 months of age with a mean ± standard error of the mean onset of 5.8 ± 0.1 months in the DQ6αβ8αβ tg strain and of 5.6 ± 0.2 months in the DQ6α8β tg strain. Female mice developed disease at a significantly earlier time points in both strains, compared with males (Figure 1). Spontaneous auricular chondritis was delayed slightly more than 1 month from the onset of polyarthritis in both strains (7.2 ± 0.4 and 7.4 ± 0.2 for DQ6αβ8αβ tg and DQ6α8β tg strains, respectively), and independent of gender. The disease was clinically not as severe as with the induced polychondritis model. These mice, however, displayed a relapsing/remitting pattern of inflammation of their ears in which the ears would become swollen and red (Figure 2) for a period of 1–2 weeks, after which the inflammation would abate for a period of 1–2 weeks. This pattern of cyclic inflammation more closely resembles the manifestation of auricular chondritis found in human RP patients. After a period of alternating relapsing and remitting phases, the ear eventually becomes a chronic 'cauliflower' ear and is reduced to a small fibrous mass as shown in Figure 2. Histological analyses of ears at various time points during these inflammatory cycles demonstrated a corresponding pattern of infiltration and a retreat of mononuclear cells during acute and remitting phases (data not shown).

**Figure 3:**
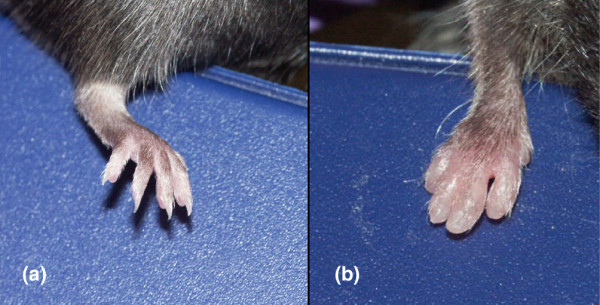
Representative photographs of polyarthritis in DQ6α8β transgenic expressing mice. **(a) **A normal rear mouse paw and **(b) **an acute arthritic rear mouse paw, representative of polyarthritis occurring during spontaneous polychondritis.

**Figure 4:**
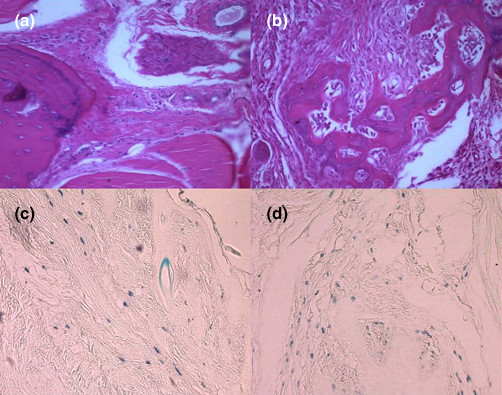
Histological assessment of arthritic synovium sections from spontaneous polychondritis and type II collagen-induced polychondritis. Sections obtained from several arthritic rear paws were stained either with **(a)**, **(b) **hematoxylin and eosin to determine the general cellular infiltration or with **(c)**, **(d) **Toludine Blue to determine the mast cell infiltration. The cellular infiltration during (a), (c) spontaneous polychondritis is compared with (b), (d) type II collagen-induced polychondritis. All sections are displayed at 10 ×.

The digits, the paws and the distal joints were affected in both DQ6αβ8αβ tg mice and DQ6α8β tg mice displaying an arthritic state with redness and swelling, compared with young or middle-aged nonarthritic DQ6α8β tg expressing mice (Figure 3). The severity of polyarthritis was equivalent in both strains with SP, and was comparable with the polyarthritis observed in induced polychondritis (data not shown). Histological examination of sections of arthritic joints of the middle-aged spontaneous mice demonstrated a correlation between cellular infiltration, predominantly mononuclear cells including mast cells (Figure 4), and the severity of polyarthritis. Sections from joints from young, nondiseased mice and from middle-aged, nonarthritic mice demonstrated normal joint architecture with no signs of inflammation or infiltration of cells. Interestingly, the level of inflammation and the numbers of cellular infiltrates appeared to be lower in spontaneous mice than that observed in collagen-immunized mice (Figure 4).

**Figure 5:**
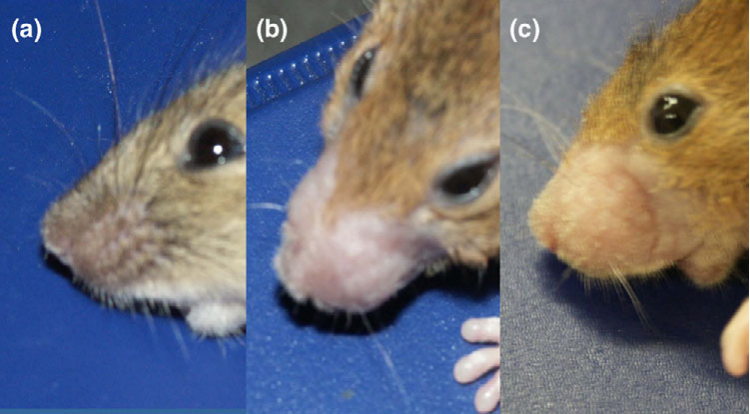
Nasal chondritis during spontaneous polychondritis in DQ6α8β transgenic expressing mice. Representative **(a) **uninflamed and **(b)**, **(c) **inflamed noses from mice with spontaneous polychondritis. Redness, swelling and accompanying hair-loss was observed in approximately 23% of the DQ6α8β transgenic expressing mice observed.

A small percentage (<25%) of the DQ6α8β tg expressing mice with SP also displayed inflamed noses (Figure 5). Histological analysis has shown that there is a massive infiltration of mononuclear cells (data not shown). Eyes of mice with SP were compared with age-matched unaffected animals and were examined for ocular involvement, including conjunctivitis, keratitis, episcleritis or uvetis associated with RP. No changes were observed that correlated with disease, although some changes related to general aging were noted (data not shown). The kidneys were also examined histologically and no cellular infiltrate was observed (data not shown).

Previous studies in human rheumatoid arthritis and RP patients and the collagen-induced arthritis models have shown antibody responses to a variety of collagens, particularly CII [5,7], as well as cartilage matrix proteins such as matrilin-1 [8], especially during the acute phase of the disease. As CII-induced murine models of both RP and rheumatoid arthritis are dependent on both exogenous CII and adjuvant, it was of particular interest whether antibody levels were elevated in SP and what antibody specificities were present in the lack of the strong but artificial CII-specific response.

**Figure 6:**
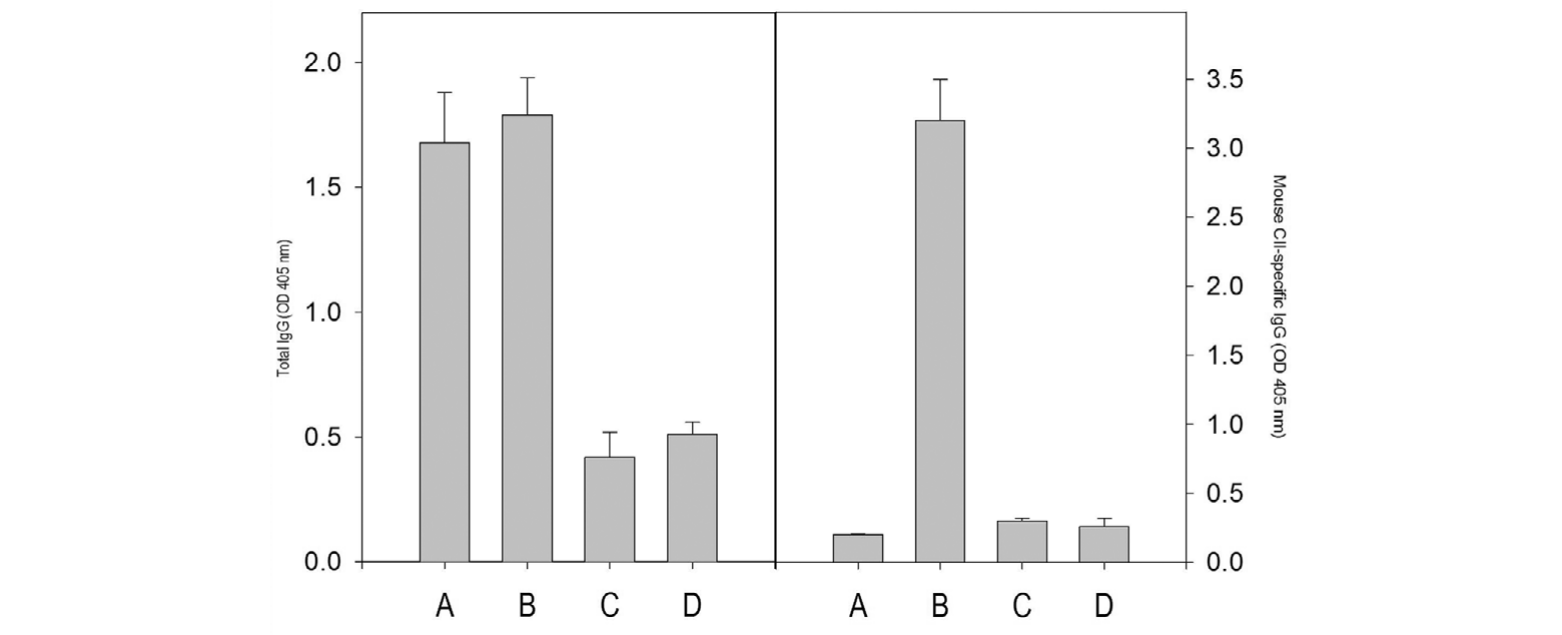
Total and type II collagen-specific IgG levels in DQ6α8β transgenic expressing mice. Sera samples from **(a) **10 middle-aged mice with spontaneous polychondritis, **(b) **from 10 young mice with type II collagen (CII)-induced polychondritis, **(c) **from 10 young naïve mice not displaying clinical disease or **(d) **from 10 middle-aged mice not displaying clinical disease were collected and assessed for the levels of total IgG and CII-specific IgG by ELISA. Polychondritic mice had greatly elevated levels of total IgG, but only CII-induced polychondritic mice had elevated CII-specific IgG. OD, optical density.

ELISAs were performed to determine total and anti-CII-specific IgG antibody levels during the disease course. All mice displaying SP also displayed a significant increase in total IgG levels, similar to the total IgG response observed in induced polychondritis in young CII-immunized mice (Figure 6). None of the spontaneous mice, however, displayed any detectable CII-specific antibodies (Figure 6). To determine whether mice developing SP displayed an antimatrilin-1 antibody response, as detected in RP patients [8], matrilin proteins were separated by SDS-PAGE, were transferred to a polyvinylidene difluoride membrane and were blotted with DQ6αβ8αβ tg experimental sera.

**Figure 7:**
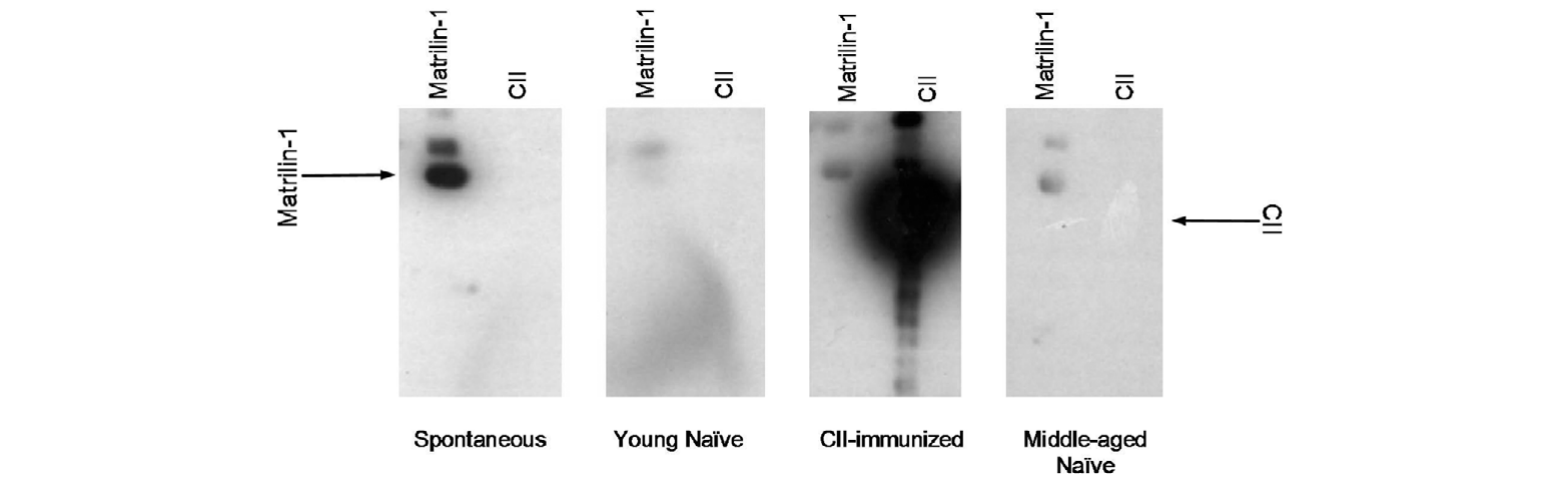
Matrilin-1-specific IgG present during spontaneous polychondritis in DQ6α8β transgenic mice. Representative sera from middle-aged mice displaying spontaneous polychondritis, from young naïve mice, from young mice with type II collagen (CII)-induced polychondritis or from middle-aged mice not displaying clinical disease were separated by SDS-PAGE electrophoresis and were blotted for the presence of matrilin-1-specific or CII-specific IgG. Although minor levels of matrilin-1-specific IgG were detectable in each sera that increased with age, it was highly elevated in each spontaneous polychondritis mouse examined. CII-specific IgG was only detected in the CII-immunized mouse, as also observed by ELISA.

Figure 7 shows a representative blot in which sera from mice were blotted against either matrilin proteins or CII. A strong IgG response to matrilin-1 was detected in sera from mice developing SP, but these mice lacked any CII-specific IgG. Conversely, young, CII-immunized DQ6αβ8αβ tg sera displayed antibodies to CII but lacked significant antibodies to matrilin-1. Sera from middle-aged DQ6αβ8αβ tg mice not exhibiting any clinical signs of polychondritis did have low levels of matrilin-1-specific IgG, yet significantly lower than those with SP. These data suggest that CII is probably not involved in the autoimmune reaction responsible for SP, and suggest that matrilin-1 may be at least one of the putative autoantigens involved in either the induction or progression of SP disease.

## Discussion

Few spontaneous models of arthritic disease have been observed [14-16], and none have as yet described all the manifestations necessary to be considered a spontaneous model of relapsing polychondritis – which is characterized not only by polyarthritis, but also by involvement of the ears, the nose and possibly the trachea. Hansson and colleagues have described a chondritic model in which matrilin-1 immunization induces involvement of the respiratory tract but in which peripheral joints and ears are unaffected [11].

We describe herein a model of spontaneous autoimmune polychondritis that occurs in mice expressing the DQ6α8β transgene without the exogenous stimulation by antigen or adjuvant and the possible interference those substances may add. The mean onset of disease occurs about 6 months of age. Although difficult to directly correlate mouse years with human years, this places the onset of disease approximately at middle age, or near the fourth or fifth decade of life in human years, matching the mean onset of RP in humans. There is a strong female preponderance, which correlates with some reports of human relapsing polychondritis [1] and autoimmune diseases in general. This would suggest that the gender-related factors that make females more prone to autoimmune responses may also be occurring in this newly identified model, and thus may be better understood. It is of note that enhanced female susceptibility is not observed in the CII-induced model of polychondritis or arthritis.

Human studies have shown that as many as two-thirds of RP patients do not develop antibodies to CII during the course of disease [17], correlating to the finding that there was not a CII-specific immune response during SP. There are, however, significant total IgG responses in both SP and RP, suggesting a role for antibodies in the pathogenesis of disease. In addition, there is an increased infiltration of mast cells into the synovium and ears during SP, which also correlates with other recent reports suggesting a pathogenic role for the elevated IgG through mast cells in RA and animal models of arthritis [18-21]. We are currently investigating the contribution the mast cells infiltrating into the cartilaginous tissues have toward the pathogenesis of SP. In addition, a strong antibody response to matrilin-1 is observed during SP, raising the possibility that matrilin-1 may be the putative autoantigen in the initiation of SP. This would correspond with an antimatrilin-1 antibody response observed in human RP patients [8] and with the ability of heterologous matrilin-1 to induce a polychondritis-like disease in mice [11].

There has been significant interest in another putative autoantigen, the ubiquitous protein glucose-6-phosphate-isomerase (G6PI), because antibodies specific for G6PI have been found deposited in the joints of RA patients as well as in mouse models of RA [22]. There have not, however, been any reports of auricular chondritis in K/B × N mice or of anti-G6PI antibodies in RP patients. Both DQ6α8β tg expressing strains displayed high titers of anti-G6PI IgG_1 _regardless of the presence of clinical disease (Bradley and Wooley, personal communication), suggesting that while the deposition of antibody complexes into the joint may contribute to the overall destruction of synovial or auricular tissue, it is most probably not the initial trigger in pathogenesis of autoimmune damage in SP.

Treatment with transforming growth factor beta (TGF-β) has been shown recently to provide some protective effect against collagen-induced arthritis in young, susceptible mice, although the effects are not long-lasting. In aging mice, however, treatment with TGF-β has been shown to block induction of arthritis altogether [23]. DQ6α8β tg expressing mice displayed an overall increase in the levels of TGF-β, regardless of the presence of disease, as compared with controls (naïve B10.T(6R) mice), with many samples reading high and out of range (Bradley and Cafruny, personal communication). It is unclear how this finding correlates with the immunoregulatory role of TGF-β in arthritis. It is possible, however, that the elevated TGF-β levels represent an active attempt by these mouse strains to downregulate the vigorous (auto)immune response. Alternatively, the TGF-β serum levels may represent another form of dysregulation, such as the role of TGF-β-specific antibodies in lupus-like diseases [24].

Correlations have been made between the HLA genotype and the genetic predisposition to many autoimmune diseases, including rheumatoid arthritis (HLA-DR4, DQ8), insulin-dependent diabetes mellitus (DR4, DQ8), thyroiditis (DR3) and multiple sclerosis (DR4) [25]. The occurrence of SP in a mouse expressing the DQ6α8β transgene and lacking endogenous MHC class II expression may provide insight into the potential contribution of mixed haplotype molecules to RP, and perhaps more generally to immune responses. It is of note that HLA-DR4 subtypes, some of which are linked with DQ8 due to linkage disequilibrium, have been associated with RP [2,26]. This association is true of the DR4 subtypes linked with DQ8 and the subtypes not linked with DQ8, however, suggesting that the RP HLA-D association may be more complex than the allelic association. This may also suggest that one or more mixed haplotypes molecules (e.g. DQ6α8β) may be able to present the pathogenic epitopes responsible for polychondritis.

## Conclusion

The SP model of RP described herein is a model lacking artificial stimulation or enhancement in which the immunopathogenic events underlying inflammation of the joints, the ears, the nose and perhaps other cartilaginous sites can be examined, including, but not limited to, the role of antibody and mast cells in RP and related diseases. In addition, this model should provide an excellent tool to evaluate therapeutic regimes for RP and other inflammatory autoimmune diseases. As SP occurs in strains of mice expressing the mixed haplotype DQ6α8β molecule, it may also provide insight into genetic susceptibility of diseases in which the allelic models of predisposition have not provided clarity.

## Abbreviations

BSA = bovine serum albumin; CII = type II collagen; ELISA = enzyme-linked immunosorbent assay; G6PI = glucose-6-phosphate-isomerase; PBS = phosphate-buffered saline; RP = relapsing polychondritis; SP = spontaneous polychondritis; tg = transgenic; TGF-β = transforming growth factor beta.

## Competing interests

The authors declare that they have no competing interests.

## Authors' contributions

JHB performed the studies of antimatrilin-1 responses. CSD generated the DQ tg mouse strains. JLL performed the *in vivo *studies, prepared the histological samples, and analyzed the total and CII-specific IgG levels. JLL and DSB conceived the study and drafted the manuscript.

## Figures and Tables

**Table 1:** Summary of spontaneous polychondritis in DQ6α8β transgenic mouse strains

Mouse strain	Incidence of spontaneous auricular chondritis (%)	Incidence of spontaneous arthritis (%)
DQ6αβ8αβ (*n *= 125)	31.2	80.8
DQ6α8β (*n *= 114)	31.6	75.4

Mice, allowed to age with no artificial immunostimulation either in the barrier breeding facility or in the conventional animal care rooms, were examined three times per week for the onset of polychondritis from 3 months to 1 year of age. Representative mice were euthanized and tissues were examined histologically to confirm the presence of disease. There was no difference in the disease incidence, the day of onset or the severity of disease, determined by the location of the mice.

## References

[B1] Letko E, Zafirakis P, Baltatzis S, Voudouri A, Livir-Rallatos C, Foster CS (2002). Relapsing polychondritis: a clinical review. Semin Arthritis Rheum.

[B2] Zeuner M, Straub RH, Rauh G, Albert ED, Scholmerich J, Lang B (1997). Relapsing polychondritis: clinical and immunogenetic analysis of 62 patients. J Rheumatol.

[B3] McAdam LP, O'Hanlan MA, Bluestone R, Pearson CM (1976). Relapsing polychondritis: prospective study of 23 patients and a review of the literature. Medicine (Baltimore).

[B4] Chang-Miller A, Okamura M, Torres VE, Michet CJ, Wagoner RD, Donadio JV, Offord KP, Holley KE (1987). Renal involvement in relapsing polychondritis. Medicine (Baltimore).

[B5] Ebringer R, Rook G, Swana GT, Bottazzo GF, Doniach D (1981). Autoantibodies to cartilage and type II collagen in relapsing polychondritis and other rheumatic diseases. Ann Rheum Dis.

[B6] Alsalameh S, Mollenhauer J, Scheuplein F, Stoss H, Kalden JR, Burkhardt H, Burmester GR (1993). Preferential cellular and humoral immune reactivities to native and denatured collagen types IX and XI in a patient with fatal relapsing polychondritis. J Rheumatol.

[B7] Yang CL, Brinckmann J, Rui HF, Vehring KH, Lehmann H, Kekow J, Wolff HH, Gross WL, Muller PK (1993). Autoantibodies to cartilage collagens in relapsing polychondritis. Arch Dermatol Res.

[B8] Buckner JH, Wu JJ, Reife RA, Terato K, Eyre DR (2000). Autoreactivity against matrilin-1 in a patient with relapsing polychondritis. Arthritis Rheum.

[B9] Bradley DS, Das P, Griffiths MM, Luthra HS, David CS (1998). HLA-DQ6/8 double transgenic mice develop auricular chondritis following type II collagen immunization: a model for human relapsing polychondritis. J Immunol.

[B10] Nabozny GH, Baisch JM, Cheng S, Cosgrove D, Griffiths MM, Luthra HS, David CS (1996). HLA-DQ8 transgenic mice are highly susceptible to collagen-induced arthritis: a novel model for human polyarthritis. J Exp Med.

[B11] Hansson AS, Heinegard D, Holmdahl R (1999). A new animal model for relapsing polychondritis, induced by cartilage matrix protein (matrilin-1). J Clin Invest.

[B12] Bradley DS, Broen JJ, Cafruny WA (1991). Infection of SCID mice with lactate dehydrogenase-elevating virus stimulates B-cell activation. Viral Immunol.

[B13] Lamoureux JL (2004). Relapsing polychondritis: induced vs. spontaneous. Dissertation.

[B14] Bouvet JP, Couderc J, Bouthillier Y, Franc B, Ducailar A, Mouton D (1990). Spontaneous rheumatoid-like arthritis in a line of mice sensitive to collagen-induced arthritis. Arthritis Rheum.

[B15] Nakamura K, Kashiwazaki S, Takagishi K, Tsukamoto Y, Morohoshi Y, Nakano T, Kimura M (1991). Spontaneous degenerative polyarthritis in male New Zealand black/KN mice. Arthritis Rheum.

[B16] Bardos T, Zhang J, Mikecz K, David CS, Glant TT (2002). Mice lacking endogenous major histocompatibility complex class II develop arthritis resembling psoriatic arthritis at an advanced age. Arthritis Rheum.

[B17] Foidart JM, Abe S, Martin GR, Zizic TM, Barnett EV, Lawley TJ, Katz SI (1978). Antibodies to type II collagen in relapsing polychondritis. N Engl J Med.

[B18] Corr M, Crain B (2002). The role of FcgammaR signaling in the K/B × N serum transfer model of arthritis. J Immunol.

[B19] Kakizoe E, Li SH, Kobayashi Y, Nishikori Y, Dekio S, Okunishi H (1999). Increases in mast cells and chymase in fibroproliferative paws of collagen-induced arthritic mice. Inflamm Res.

[B20] Kobayashi Y, Okunishi H (2002). Mast cells as a target of rheumatoid arthritis treatment. Jpn J Pharmacol.

[B21] Secor VH, Secor WE, Gutekunst CA, Brown MA (2000). Mast cells are essential for early onset and severe disease in a murine model of multiple sclerosis. J Exp Med.

[B22] Matsumoto I, Staub A, Benoist C, Mathis D (1999). Arthritis provoked by linked T and B cell recognition of a glycolytic enzyme. Science.

[B23] Thorbecke GJ, Shah R, Leu CH, Kuruvilla AP, Hardison AM, Palladino MA (1992). Involvement of endogenous tumor necrosis factor alpha and transforming growth factor beta during induction of collagen type II arthritis in mice. Proc Natl Acad Sci USA.

[B24] Zitterkopf NL, Jones QA, Bradley DS, Durick K, Rowland RR, Plagemann PG, Cafruny WA (2003). Hydrophobic IgG-containing immune complexes in the plasma of autoimmune MRL/lpr mice, lactate dehydrogenase-elevating virus-infected mice, and pigs: association with transforming growth factor-beta and pH-dependent amplification. Viral Immunol.

[B25] Taneja V, David CS (1999). HLA class II transgenic mice as models of human diseases. Immunol Rev.

[B26] Lang B, Rothenfusser A, Lanchbury JS, Rauh G, Breedveld FC, Urlacher A, Albert ED, Peter HH, Melchers I (1993). Susceptibility to relapsing polychondritis is associated with HLA-DR4. Arthritis Rheum.

